# Efficacy of Oral Vitamin K2 Supplementation in Experimental Knee Osteoarthritis

**DOI:** 10.3390/metabo16060425

**Published:** 2026-06-17

**Authors:** Emre Uzun, İbrahim Tekeoğlu, Hüseyin Çakıroğlu, Özcan Budak, Elvan Şahin, Kemal Nas, Muhammed Zahid Sahin, Ayhan Kamanlı

**Affiliations:** 1 Department of Physical Medicine and Rehabilitation, Sakarya Training and Research Hospital, Sakarya 54290, Turkey; 2 Department of Physical Medicine and Rehabilitation, Kutahya Health Sciences University, Kutahya 43020, Turkey; ibrahim.tekeoglu@ksbu.edu.tr; 3 Experimental Animal Unit, Faculty of Medicine, Sakarya University, Sakarya 54290, Turkey; huseyincakiroglu@sakarya.edu.tr; 4 Department of Histology and Embryology, Faculty of Medicine, Sakarya University, Sakarya 54290, Turkey; ozcanbudak@sakarya.edu.tr (Ö.B.); elvansahin@sakarya.edu.tr (E.Ş.); 5 Department of Physical Medicine and Rehabilitation, Faculty of Medicine, Sakarya University, Sakarya 54290, Turkey; kemalnas@sakarya.edu.tr (K.N.); zsahin@sakarya.edu.tr (M.Z.S.)

**Keywords:** osteoarthritis, vitamin K2, monosodium iodoacetate, menaquinone, menadione, rat model

## Abstract

**Background/Objectives**: Although vitamin K has been implicated in osteoarthritis pathophysiology, the specific effects of vitamin K2 (menaquinone) on cartilage degeneration remain poorly characterized. This study aimed to investigate the effect of oral vitamin K2 supplementation in a monosodium iodoacetate-induced osteoarthritis model. **Methods**: Twenty-four male Sprague Dawley rats were included in the study and divided into 3 equal groups: sham group, control (osteoarthritis) group, and treatment group. Saline was applied to the right knee of the sham group, and MIA was applied intra-articularly to the right knee of the control and treatment groups to create an osteoarthritis model. Rats in the treatment group were given 8 micrograms (μg)/day of vitamin K2 orally in addition to the standard diet. After 28 days of follow-up, all rats were euthanized. The right knee articular cartilage was examined histologically with Hematoxylin–Eosin and Safranin O and immunohistochemically with type II collagen alpha 1 and Matrix Metalloproteinase-13. **Results**: Histological evaluation demonstrated significantly lower Mankin scores in the treatment group (4.25 ± 0.83) compared with the control group (11.10 ± 0.83). Immunohistochemical analysis showed more intense type II collagen staining and reduced matrix metalloproteinase-13 staining in the treatment group relative to the control group. **Conclusions**: Oral vitamin K2 administration was associated with reduced cartilage degeneration and improved matrix preservation at the 28-day endpoint in an induced MIA osteoarthritis rat model.

## 1. Introduction

Osteoarthritis is a joint disorder characterized by cellular stress and extracellular matrix degradation triggered by micro- and macro-injuries, leading to maladaptive repair and pro-inflammatory responses. It is most commonly observed in the knee joint [1] and affects not only the articular cartilage but also the subchondral bone, ligaments, capsule, synovium, and muscles [2]. Morphological changes include cartilage irregularities, thinning, superficial cracks, and exposure of the underlying bone. As the condition advances, marginal osteophytes form, which are covered by hyaline cartilage and fibrocartilage [3].

Vitamin K is a group of fat-soluble vitamins existing in three main forms: K1 (phylloquinone), K2 (menaquinone-MK), and K3 (menadione). Vitamin K1 is well known for its role in coagulation factors [4]. Vitamin K2 plays a critical role in bone health by activating markers like osteocalcin, promoting osteoblastic activity, and being used in osteoporosis treatment [5]. It also regulates collagen cross-linking [6]. Vitamin K3 is a synthetic form [4].

Subclinical vitamin K deficiency has been associated with increased knee OA and cartilage lesions [7]. Serum vitamin K deficiency in elderly men has been correlated with meniscus damage and cartilage loss [8]. Additionally, gross osteophyte formation has been linked to vitamin K deficiency [9].

Recent studies [10,11] have highlighted that the effects of vitamin K on bone metabolism are primarily mediated through K2. In patients undergoing total knee arthroplasty, K2 vitamin levels in the medial femoral and tibial condyles were found to be lower than those in lateral condyles, aligning with the clinical knowledge that OA predominantly affects the medial side [12].

This study aims to investigate the effects of vitamin K2 (menaquinone) on knee OA, a progressively disabling condition.

## 2. Materials and Methods

All animal experiments were conducted in accordance with the ARRIVE guidelines and complied with the EU Directive 2010/63/EU for the protection of animals used for scientific purposes. The study was approved by the Institutional Animal Ethics Committee (Decision No. 06, dated 12 January 2022).

### 2.1. Animals and Experimental Design

The study utilized 14-week-old male Sprague Dawley rats weighing 250–330 g. Using the study by Alibegovic et al. [13], the total sample size required was calculated to be 24 (*n* = 8 per group) using the G-Power software (version 3.1.9.7, Heinrich Heine University, Düsseldorf, Germany) package with an effect size of 0.90, 95% power, and a margin of error of 0.05. A simple randomization method was used to allocate animals into three groups (sham group, control group, and treatment group). Group assignments were generated using a computer-based random number generator by an independent researcher who was not involved in the experimental procedures. Allocation was concealed from the investigators performing the injections and daily follow-up to minimize selection bias. The knee OA model was induced using Monosodium Iodoacetate (MIA) obtained from Sigma Aldrich (Schnelldorf, Germany). To create an OA model, rats were anesthetized intraperitoneally. After anesthesia, the right patellar tendon of the rats was palpated, and intra-articular intervention was performed with a 26-gauge needle to the medial aspect of the tendon.

1. Sham Group (*n* = 8): Under intraperitoneal anesthesia, the right knee femorotibial joint was injected with 50 μL of 0.9% physiological saline. In addition to a standard diet, the group received 2 μL oral saline daily using a calibrated micropipette.
2. Control Group (OA Group, *n* = 8): Under intraperitoneal anesthesia, the right knee femorotibial joint was injected with 3 mg MIA dissolved in 50 μL of 0.9% physiological saline. In addition to a standard diet, the group received 2 μL oral saline daily using a calibrated micropipette.
3. Treatment Group (OA + Oral K2 Vitamin Group, *n* = 8): Under intraperitoneal anesthesia, the right knee femorotibial joint was injected with 3 mg MIA dissolved in 50 μL of 0.9% physiological saline. The group received 8 μg/day vitamin K2 orally in a volume of 2 μL using a calibrated micropipette, in addition to a standard diet.

The treatment group received 8 μg/day vitamin K2 orally. The sham and OA control groups received an equivalent volume of saline to control for the effects of oral administration. Since no specific K2 vitamin dose for OA treatment exists in the literature, the dose of 8 μg/day, commonly used in osteoporosis studies, was selected [14]. The vitamin K2 preparation used in this study was Venatura^®^ (Istanbul, Turkey), a commercial oral drop formulation containing a combination of 80% MK-4 and 20% MK-7.

The animals were housed in separate cages under identical environmental conditions (e.g., temperature, humidity, and light/dark cycles). The location of the cages within the facility was rotated weekly to eliminate the potential influence of cage placement on the results. Treatments and measurements were performed in a randomized order.

During the experimental process, the animals were monitored daily and recorded for potential adverse events. Body weights were measured weekly. Animals exhibiting significant weight loss, signs of infection, marked reduction in food and water intake, persistent immobility, or deviation from normal behavior were planned to be excluded from the study.

### 2.2. Data Collection and Analysis

#### 2.2.1. Histological Analysis

After 28 days, the rats were sacrificed, and their right knee femorotibial joints were carefully extracted. Tissue samples were fixed in 10% neutral formaldehyde solution and then decalcified in 10% EDTA solution (pH 7.2–7.4) for 21 days. The EDTA solution was replaced every three days throughout the decalcification process to ensure optimal efficiency. After decalcification, the specimens were washed with running water, dehydrated through graded alcohol solutions, cleared with xylene, and embedded in paraffin. Sections 3 μm thick were meticulously stained with Hematoxylin–Eosin and Safranin-O [14,15].

#### 2.2.2. Immunohistochemical Analysis

For immunohistochemical analysis, 3 μm sections were deparaffinized and rehydrated through a graded alcohol series. Antigen retrieval was performed using citrate buffer (pH 6). Sections were washed with phosphate-buffered saline and blocked for peroxidase activity using 3% hydrogen peroxide. Protein block was applied, followed by incubation with anti-type II collagen alpha-1 (Biorbyt^®^ orb683078, Cambridge, UK) and Matrix Metalloproteinase-13 (MMP-13-Biorbyt^®^ orb11057, Cambridge, UK) antibodies for 2 h. The sections were sequentially treated with Primary Antibody Amplifier Quanto and HRP Polymer Quanto. Final staining was performed with 3,3′-Diaminobenzidine, and counterstaining was achieved using hematoxylin. Prepared slides were cleaned with xylene and mounted with Entellan. For quality assurance, negative control sections were processed at the same time under identical conditions, with the exception that the primary antibody was omitted and replaced by antibody diluent. No specific staining was observed in these negative control sections.

#### 2.2.3. Histopathological Assessment

Histopathological evaluation was conducted using the Mankin scoring system [16]. Immunohistochemical staining was quantified as the percentage of positive staining in 200 randomly selected cells, including hotspot areas [17]. Positive staining ratios were calculated as the percentage of total positively stained cells. Immunohistochemical staining scoring was performed using a scoring algorithm calculated with the H-Score formula [18].

Two independent histologists, blinded to all group assignments, performed the histopathological evaluations and scoring. The final score for each specimen was derived from the mean of their assessments.

#### 2.2.4. Statistical Analysis

The distribution of variables was assessed using the Kolmogorov–Smirnov test. For comparisons among the three independent groups, the Kruskal–Wallis test was used. When an overall intergroup difference was detected, pairwise comparisons between independent groups were performed using the Mann–Whitney U test with Bonferroni correction to adjust for multiple comparisons. A *p*-value of <0.05 was considered statistically significant.

## 3. Results

All animals successfully completed the experimental protocol, and no procedure-related mortality or adverse effects were observed during the 28-day follow-up period.

### 3.1. Analysis of Histopathological Data and Mankin Scores of Knee Joints

**Figure 1:**
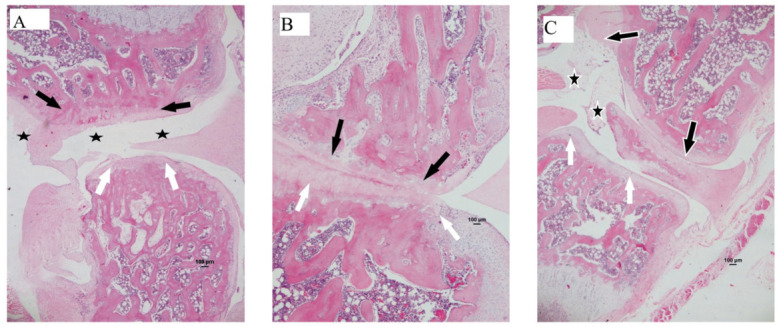
Representative histological images of knee joints stained with Hematoxylin and eosin. (**A**) Sham group, (**B**) Control (OA) group, (**C**) Treatment group. (Black arrows indicate the femoral articular cartilage, white arrows indicate the tibial articular cartilage, and black stars indicate the joint space) Scale bar = 100 μm.

Histological examination with Hematoxylin–Eosin staining revealed preserved joint cartilage structure in the sham group. The cartilage tissue, underlying bone, and bone marrow structures were preserved without histopathological alterations (Figure 1A).

In the control (OA) group, synovial tissue thickening was prominently observed. Compared to the sham group, the cartilage tissue in the control group showed thinner regions. Histological preparations predominantly displayed areas where the cartilage had thinned, and the underlying bone tissue was denser. Synovial tissue debris in the joint space was also easily noticeable (Figure 1B).

In the treatment group, histological areas showing preserved articular cartilage and bone structure were observed. Synovial tissue debris was not detected in the joint space. Compared to the control (OA) group, there were fewer degenerated cartilage areas, and some thickened cartilage tissue regions were identified (Figure 1C).

**Figure 2:**
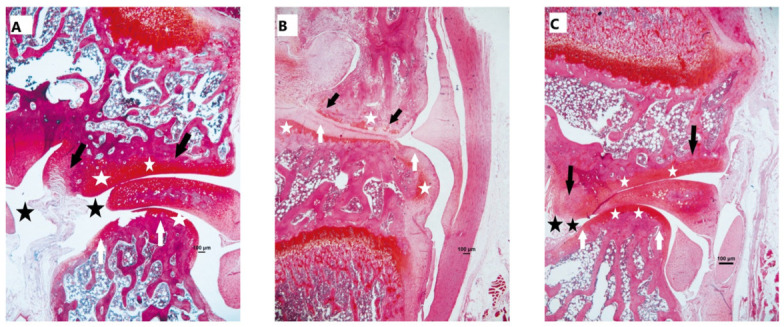
Representative images showing Safranin O staining of knee joint cartilage. (**A**) Sham group, (**B**) Control (OA) group, (**C**) Treatment group. (Black arrows indicate the femoral articular cartilage, white arrows indicate the tibial articular cartilage, black stars indicate the joint space, and white stars indicate the proteoglycan-rich cartilage matrix (Safranin O-positive) Scale bar = 100 μm.

Histological examination with Safranin-O staining revealed intense staining of cartilage tissue in the sham group (Figure 2A). In the control (OA) group, staining was significantly reduced compared to the sham group (Figure 2B). In the treatment group, staining was more intense compared to the control (OA) group (Figure 2C) [19].

Mankin scores were significantly lower in the treatment group compared to the control (OA) group, indicating reduced cartilage degeneration (*p* < 0.05). Pairwise comparisons with Bonferroni correction confirmed significant differences among all groups (all adjusted *p* < 0.05) (Table 1).

### 3.2. Immunohistochemical Analysis of MMP-13 and Type II Collagen Alpha-1 Antibody Staining in Knee Joints

**Figure 3:**
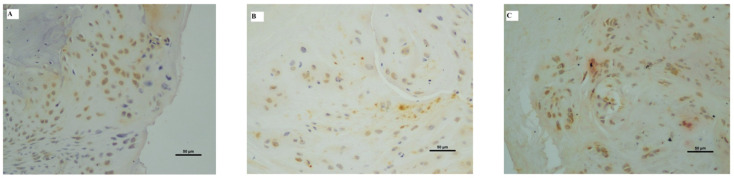
Representative immunohistochemical images of MMP-13 staining in knee joint tissue. (**A**) Sham group, (**B**) Control (OA) group, (**C**) Treatment group. Brown staining indicates regions positive for MMP-13 immunoreactivity. Scale bar = 50 μm.

Immunohistochemical examination with MMP-13 antibody staining revealed more intense staining in the control group compared to the sham group (Figure 3A,B). In the treatment group, staining with MMP-13 antibody was less intense compared to the control (OA) group (Figure 3A,C).

**Figure 4:**
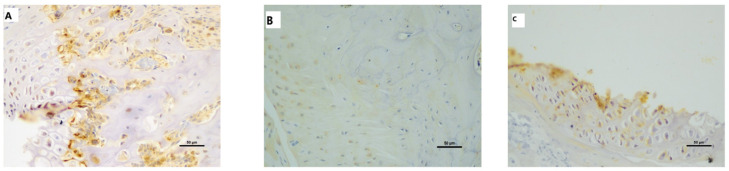
Representative immunohistochemical images of type II collagen alpha 1 staining in knee joint tissue. (**A**) Sham group, (**B**) Control (OA) group, (**C**) Treatment group. (Brown staining indicates regions positive for type II collagen alpha 1 immunoreactivity) Scale bar = 50 μm.

For type II collagen alpha-1 antibody staining, reduced staining was observed in the control (OA) group compared to the sham group (Figure 4A,B). In the treatment group, staining was more intense compared to the control (OA) group (Figure 4B,C).

Type II collagen immunoreactivity (H-score) was significantly higher in the treatment group compared to the control group (35.00 ± 7.60 vs. 6.30 ± 2.31). Similarly, MMP-13 immunoreactivity (H-score) was significantly lower in the treatment group (28.80 ± 8.30 vs. 71.30 ± 2.31). These findings indicate reduced cartilage degradation and preservation of extracellular matrix in the treatment group. Pairwise comparisons with Bonferroni correction confirmed that these differences remained statistically significant (adjusted *p* < 0.05) (Table 2).

## 4. Discussion

In the present study, oral vitamin K2 supplementation significantly attenuated cartilage degeneration in a monosodium iodoacetate-induced osteoarthritis rat model. Histopathological analyses revealed markedly lower Mankin scores, accompanied by preservation of type II collagen and suppression of MMP-13 expression in the treatment group compared with untreated osteoarthritic controls. These findings suggest that vitamin K2 exerts a protective effect on articular cartilage integrity, potentially by modulating extracellular matrix turnover and limiting collagen degradation. Because vitamin K2 administration was initiated in parallel with MIA induction, these findings should be interpreted within the context of an experimental OA model rather than as evidence of long-term disease modification.

OA is one of the most prevalent musculoskeletal disorders in humans and animals [20]. Although studies on its pathogenesis and treatment are ongoing, there is currently no treatment that halts or reverses the disease. In this study, a human OA-like condition was induced in rats through MIA injection [21]. Literature shows that MIA effectively induces physical and histological findings associated with OA within 28 days [22,23,24]. In our study, oral vitamin K2 treatment was initiated on the same day as the MIA injection.

The relative efficacy of MK-7 and MK-4, subtypes of vitamin K2, remains unclear. MK-7 has higher bioavailability compared to other K2 vitamin forms. Studies on healthy individuals [25,26] have shown that MK-7 achieves detectable serum levels more rapidly. In a study [27] investigating the inhibition of NF-κB activation, MK-7 was found to be 10 times more effective than MK-4. The lethal dose for a single dose of vitamin MK-7 was observed to be higher than 2000 mg/kg [28]. Additionally, no toxicological findings were observed in mice given MK-7 at a dose of 10 mg/kg for 90 days [28]. It should be noted that the preparation used in the present study was not an isolated MK-4 or MK-7 formulation, but a commercial combination product containing 80% MK-4 and 20% MK-7. Therefore, the present findings should be interpreted in the context of this combined oral vitamin K2 preparation.

Our findings indicate that the group receiving vitamin K2 exhibited reduced histological and immunohistochemical changes associated with OA. Increased type II collagen alpha-1 staining and decreased MMP-13 staining in the vitamin K2-treated group compared to the control group provide strong evidence of the anti-osteoarthritic effects of vitamin K2 in experimentally induced acute OA.

The pathogenesis of OA is complex and multifaceted. Based on current literature, several mechanisms may underlie the potential anti-OA effects of vitamin K2. The extracellular matrix (ECM) of articular cartilage contains proteoglycans, collagen, elastic and reticular fibers, and various glycoproteins [29]. In cartilage undergoing OA, the amount of proteoglycan decreases, and its structure deteriorates. As a result, mechanical stress causes a direct load on the collagen. The collagen most affected by this situation is type II collagen [30]. Although collagen is resistant to most proteases, it is recognized especially by MMP-13 and is hydrolyzed and degraded. For this reason, while the amount of type II collagen is decreased in joint cartilage with OA, an increase in the level of MMP-13 is detected [31]. The activity of MMP-13 is affected by many factors. It has been reported that fibroblast growth factor-2 [32], bone morphogenetic protein-2 (BMP-2) [33], and NF-κB signaling pathway activation [34] increase MMP-13 expression. Additionally, TNF-α has been shown to elevate MMP-13 levels in chondrocytes [35,36]. Vitamin K2 is known to inhibit TNF-α and NF-κB, which may explain the downregulation of MMP-13 observed in articular cartilage. However, activation of BMP-2 and Runx2 could also enhance MMP-13 expression [37,38]. These data suggest that other factors may be involved in the downregulation of MMP-13 by vitamin K2. Thus, the molecular mechanisms involved require further investigation.

Osteoblast dysregulation has been implicated in OA pathogenesis, particularly through altered RANKL/OPG balance, prostaglandin E2 (PG-E2) signaling, and osteophyte formation [39,40,41]. Vitamin K2 has been shown in other experimental models to influence osteoblast differentiation and PG-E2 production, raising the possibility that it may modulate subchondral bone remodeling in OA [40]. Calcification of articular cartilage and soft tissues is another factor associated with OA progression. Vitamin K-dependent proteins such as matrix Gla-protein (MGP) and Gla-rich protein (GRP) require vitamin K-mediated carboxylation to inhibit pathological mineralization [42]. Prior studies have shown that inadequately carboxylated MGP and GRP are associated with more severe OA changes [43].

**Figure 5:**
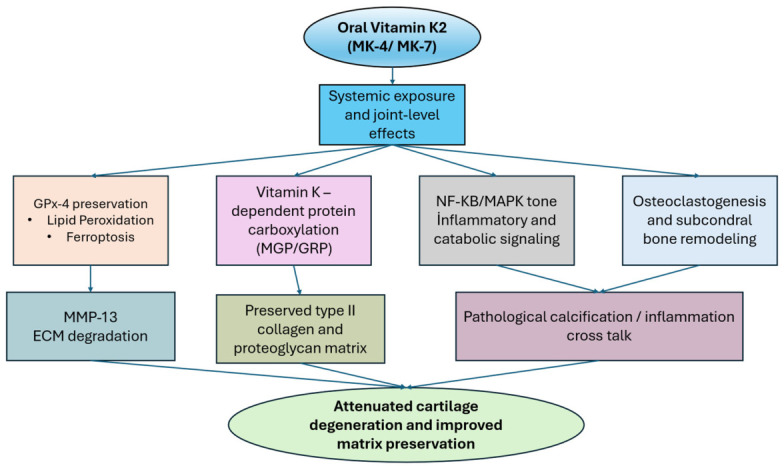
Proposed mechanism by which oral vitamin K2 may attenuate experimental osteoarthritis. The scheme integrates evidence from K2/GPX4-mediated anti-ferroptotic effects, vitamin K-Gas6/AXL signaling, vitamin K-dependent matrix Gla protein/Gla-rich protein carboxylation, NF-κB regulation, and subchondral bone remodeling pathways.

Vitamin K2 may reduce OA-related cartilage damage through converging pathways: preservation of GPX4 activity and redox balance may limit chondrocyte ferroptosis, lipid peroxidation, and downstream MMP-13-driven matrix degradation [44,45,46]. Vitamin K-dependent gamma-carboxylation may enhance the functional activity of matrix Gla protein and Gla-rich protein, thereby modulating pathological calcification and the calcification-inflammation crosstalk within joint tissues [42,43,47,48], and MK-4/MK-7-related regulation of NF-κB signaling and osteoclastogenesis may indirectly influence subchondral bone remodeling and the whole-joint degenerative process [27,49]. Plausible mechanisms linking oral vitamin K2 to the observed histological improvement are summarized in Figure 5. However, because GPX4, Gas6/AXL, MGP/GRP carboxylation status, synovial inflammation, and subchondral bone parameters were not directly measured, these pathways should be regarded as biologically plausible mechanisms rather than confirmed causal mediators in the present experiment.

Although prior studies have explored vitamin K-related pathways in osteoarthritis, research evaluating oral vitamin K2 supplementation in experimentally induced OA remains limited. Our study contributes to this literature by providing in vivo data in an MIA-induced rat model. These results add further support to the emerging evidence that vitamin K2 may modulate OA progression. To the best of our knowledge, this is the first in vivo study to demonstrate that oral vitamin K2 monotherapy can attenuate histological cartilage degeneration in an experimentally induced OA model. This distinction is important because the closest available evidence differs from our design in route, treatment context, or model structure. He et al. reported beneficial effects of intra-articular MK-7 in an ACLT-induced rat OA model through GPX4-mediated suppression of ferroptosis and extracellular matrix degradation [44]. Zhao et al. evaluated oral MK-4 only in combination with calcitriol in medial meniscus destabilization and osteoporotic OA mouse models, mainly linking the effect to osteoclastogenesis and subchondral bone remodeling [50]. Rudig et al. investigated chondrocytes obtained from donor rats exposed to an MK-7-enriched diet, but not an in vivo OA treatment model [49]. Recent vitamin K-Gas6 data support an anti-ferroptotic role of vitamin K in OA, although the in vivo intervention used intra-articular vitamin K1 rather than oral K2 [46]. Therefore, our findings do not merely repeat the concept that vitamin K-related pathways are involved in OA. In addition, they extend the field by showing that a clinically practical oral K2 formulation can produce measurable cartilage-protective signals in an MIA-induced OA model.

The preliminary findings of this study were previously presented at an international rheumatology congress [19]. A limitation of our study is the single 28-day endpoint, which does not allow definitive conclusions regarding long-term progression or disease-modifying effects. In addition, the pathways through which the protective effect may have occurred could not be examined in detail. Another limitation of this study is the absence of functional outcome assessments, such as pain behavior, mobility, or weight-bearing measurements, which could have provided complementary information regarding the clinical relevance of the histological and immunohistochemical findings. These analyses were not feasible within the scope of the present study and the available project resources and budget. Additionally, during the study period (June 2023–July 2023), oral vitamin K2 supplements in Turkey were generally available as combined MK-4/MK-7 formulations. Therefore, the findings of this study reflect the effects of a combined preparation rather than an isolated K2 subtype. One limitation of this study is that representative images of positive and negative immunohistochemical control sections were not archived, although appropriate control procedures were performed during the staining process. Further studies incorporating isolated K2 subtypes, broader mechanistic analyses, and functional assessments would be valuable.

Taken together, these results position oral vitamin K2 as a novel and translationally relevant experimental strategy for early cartilage protection in OA. Nevertheless, the present data should be interpreted as histological and immunohistochemical evidence of protection at a 28-day endpoint rather than definitive proof of long-term disease modification. Future studies using isolated MK-4 and MK-7 preparations, delayed-treatment designs, functional pain and gait assessments, and direct measurement of GPX4/Gas6/AXL, MGP/GRP carboxylation, synovial inflammatory markers, and subchondral bone remodeling would be needed to clarify the dominant mechanism and therapeutic relevance.

## 5. Conclusions

In this induced MIA rat model of osteoarthritis, oral vitamin K2 administration was associated with lower histological degeneration scores, reduced MMP-13 immunostaining, and better preservation of type II collagen at the 28-day endpoint. These findings suggest a potential protective effect of oral vitamin K2 on cartilage integrity in this experimental setting. Given its oral route and clinical practicality, oral vitamin K2 may represent a promising candidate for further investigation in OA-related cartilage protection. However, because the present study was limited to histological evaluation and a limited set of immunohistochemical assessments in an experimental OA model, further studies including broader mechanistic analyses, synovial tissue evaluation, and synovial fluid investigations are needed before broader conclusions can be drawn.

## Figures and Tables

**Table 1:** Comparison of histological Mankin scores among experimental groups.

Parameter	Statistic	Sham Group	Control Group	Treatment Group	*p*-Value
Mankin score	Mean ± SD	0.63 ± 0.52	11.10 ± 0.83	4.25 ± 1.16	<0.001
	Median	1.00 (0.00–1.00) ^2,3^	11.00 (10.75–12.00)	4.50 (3.00–5.00) ^2^	

*p*-values represent overall intergroup comparisons by the Kruskal–Wallis test. Pairwise differences were assessed using the Mann–Whitney U test with Bonferroni correction. Superscript ^2^ indicates significant difference versus the control group (adjusted *p* < 0.05), and superscript ^3^ indicates significant difference versus the treatment group (adjusted *p* < 0.05). Mean ± SD values are presented for descriptive purposes, whereas inferential analyses were based on non-parametric tests. Median values are presented together with interquartile range (IQR; 25th–75th percentiles).

**Table 2:** Comparison of histological immunohistochemical H-scores among experimental groups.

Parameter	Statistic	Sham Group	Control Group	Treatment Group	*p*-Value
Type II collagen	Mean ± SD	58.80 ± 5.20	6.30 ± 2.31	35.00 ± 7.60	<0.001
	Median	60.00 (55.00–61.23) ^2,3^	5.00 (5.00–6.25)	35.00 (30.00–36.25) ^2^	
MMP-13	Mean ± SD	11.50 ± 2.27	71.30 ± 2.31	28.80 ± 8.30	<0.001
	Median	10.00 (10.00–12.75) ^2,3^	70.00 (70.00–71.25)	27.50 (23.75–31.25) ^2^	

*p*-values represent overall intergroup comparisons by the Kruskal–Wallis test. Pairwise differences were assessed using the Mann–Whitney U test with Bonferroni correction. Superscript ^2^ indicates significant difference versus the control group (adjusted *p* < 0.05), and superscript ^3^ indicates significant difference versus the treatment group (adjusted *p* < 0.05). Mean ± SD values are presented for descriptive purposes, whereas inferential analyses were based on non-parametric tests. Median values are presented together with interquartile range (IQR; 25th–75th percentiles).

## Data Availability

All data supporting the findings of this study are available from the corresponding author upon reasonable request.

## Abbreviations

The following abbreviations are used in this manuscript:

BMP-2	Bone Morphogenetic Protein-2
GPX-4	Glutathione Peroxidase-4
MGP	Matrix Gla-protein
MIA	Monosodium Iodoacetate
MK	Menaquinone
MMP-13	Matrix Metalloproteinase-13
OA	Osteoarthritis
